# Medical Items and News of Interest to the Physician

**Published:** 1880-01

**Authors:** 


### Medical Items and News,

### For the Use of Physicians.

CULLED FROM THE STANDARD MEDICAL JOURNALS OF THE WORLD.

NOTE.—These formulæ are intended only for the regular practitioner of medicine, and should be employed only by him, or under his immediate supervision.

**Dr. Wheelock's Cough Mixture.**

℞

Ether. sulph., ʒ iii.
Tinct. hyociam.,
Syr. pruni virg.,
Syr. tolutan, *aa.* ℥ i.
Aquæ, ad. ℥ iv.

Dose, two to four drachms.

---

**Chloroform Cough Mixture.**

℞

Morphiæ acet., gr. iii.

Tr. belladonnæ, ʒ iv.

Spts. chloroformi, ʒ vi.

Syr. senegæ, ℥ i.

Syr. pruni virg., ad. ℥ iv.

---

**Pertussis.**

The latest specific in whooping cough is the oxalate of cerium. It is given in doses of gr. ½–2, before breakfast, once daily. Dr. T. Clark has recommended it in the *Practitioner*, and a New York physician, Dr. Morje, has reported favorably from its use.

---

**Sore Nipples.**

Chapped nipples are treated either by the glycerole of the nitrate of lead in a solution containing from twenty grains to one drachm to the ounce, or by a mixture of two drachms of iodoform to the ounce of balsam of Peru. The balsam is used because it disguises the smell of the iodoform.

---

**Goulard's Cerate.**

C. Bernbeck thinks that the cerate of subacetate of lead ought either to be discarded altogether as a healing salve or at least be made extempore, because he frequently found it to contain free acetic acid when a few days old, which, of course, makes the cerate irritating instead of healing. The presence of acetic acid can readily be determined by the odor and by triturating five grammes of the cerate in a mortar with an equal quantity of alcohol, and testing with blue litmus paper previously moistened with water.—*Pharm. Zig.*

**Acne Punctata.**

The acne which occurs on the face of young persons is often very distressing to them. Dr. J. G. Parsons tells, in the *British Medical Journal*, a simple and efficient remedy. It is to dust the face with pure precipitated sulphur every night, with an ordinary toilet puff. This will usually effect a cure in about a week.

---

**Sydenham's Anti-Spasmodic Mixture.**

℞

Tincture of valerian, 5 parts.
Tincture of castoreum, 10 "
Hoffman's anodyne, 1 "
Fennel water, 100 "

M. A tablespoonful every three or four hours.

---

**Conical Cervix.**

This condition is treated either by forcible dilatation with a strong uterine dilator, or by lateral section with a hysterotome. If the cervix is sickle-shaped, a section of the posterior lip is performed. The subsequent treatment consists in such local applications as tend to keep the parts from closing up.

---

**Anti-Asthmatic Powder.**

The *Practicien* gives the following formula, which has been adopted with much success by Dr. de Crevoisier, of Briey. Take equal weights of stramonium, sage, belladonna, and digitalis; crush to about the coarseness of sawdust, damp a little, and mix in as much nitre as of one of the other substances. Burn a little on a plate, cover with a paper cone open at the top, and let the sufferer inhale the smoke. If the smoke is too abundant, damp the mixture with a little water.

---

**Hoarseness.**

Borax and nitrate of potassium have been employed with advantage in cases of hoarseness and aphonia occurring suddenly from the action of cold. The remedy is recommended to singers and orators whose voices suddenly become lost, but which by these means can be recovered almost instantly. A little piece of borax, the size of a pea, is to be dissolved in the mouth, ten minutes before singing or speaking ; the remedy provokes an abundant secretion of saliva, which moistens the mouth and throat. This local action of the borax should be aided by an equal dose of nitrate of potassium, taken in warm solution, before going to bed.—*La France Medicale.*

**Antiseptic Balsam.**

℞

| | | |
|---|---|---|
| Acidi carbolici puri, | partes | 4. |
| Morphiæ muriatis, | " | 1. |
| Tr. arnicæ, | " | 10. |
| Tr. aconiti, | " | 10. |
| Balsami Peruviani, | " | 25. |
| Glycerinæ, | " | 50. |

Mix.

**Gout.**

From his experience in a number of cases, Dr. C. W. Schœneman (*Pacific Med. Journal*) believes that quinine will cut short an attack of gout. He gives:

℞

Quiniæ sulphatis, gr. iij.

Sodii bicarb, gr. xij.

M. This amount every two hours during the day.

No other medication is needed, though the joint may be painted with tincture of iodine.

**Diphtheritic Poison.**

A singular instance of the vitality of the poison of diphtheria is reported in the *Vratschebnyia Vedomosti*. A gentleman in the south of Russia had, four years ago, lost a boy from diphtheria. A family vault having recently been constructed, the coffin of the boy was transferred thither. Before it was lowered down into the vault, the father wished to look at the body, having entertained a suspicion that the child had been buried alive. An opening was accordingly made in the lid of the coffin, the whole family, including five children, looking on. The next day all the children were ill with diphtheria, and one of them has since died.

**Carbuncle of the Urethra.**

In timid women who refuse to submit to an operation, either mummify the growth with crystalized carbolic acid melted down by heat, or destroy it by applications of chromic acid made with the utmost care, by means of a match whittled down to a point. The excess of acid is afterward neutralized by means of injections of a strong solution of the bicarbonate of sodium. If an operation is permitted, cut off the growth with a pair of scissors curved on the flat, and sear the wound with a hot iron wire, or with Paquelin's *Thermo-cautere*. The use of an alcohol lamp is advised for heating the wire, because, when an ordinary light is used, the impression upon the operator's retina, made by the bright flame, so obscures his vision that the wire grows cool before he can clearly see the point where the application is to be made. He hastens the healing of the cauterized surface by occasional applications of carbolic acid, or by dusting it with iodoform.

**For Freckles.**

℞

Sulphophenate of zinc, 1 part.

Oil of lemon, 1 part.

Pure alcohol, 5 parts.

Collodion, 45 parts.

Mix well together by trituration.

**Hæmatoma of Douglas's Pouch.**

The patient is kept absolutely quiet, and astringent drinks, such as sulphuric acid, lemonade, etc., administered. Opium enough is given to lull the pain and keep the patient thoroughly quiet. For a number of hours following the attack but very little food is given. Stimulants are avoided. Occasionally the vagina is packed with ice.—*Dr. Goodell's Clinic.*

**Gathered Breasts.**

When an abscess forms in the mammary glands, an early incision is practiced. If the pus lies deep or is lodged behind the gland, a cutaneous incision is first made, a grooved director is then pushed into the abscess, and the opening is enlarged by the uterine dilator. The breast is then tightly strapped with adhesive plaster, and treated by a dry compress of oakum. Should the abscess show symptoms of becoming chronic, its walls are overdistended by an injection of a three per cent. solution of carbolic acid. This overstretching is practised so that the acid may reach every nook and cranny of the purulent cavity.—*Ibid.*

**Vaginitis.**

Non-specific and acute cases of vaginitis are treated by such hot and emollient injections as flaxseed, or slippery-elm bark tea, to which laudanum has been added; the solution which is usually employed contains laudanum f. ℥ ij. to Oij. of flaxseed. When the inflammation has subsided, vaginal suppositories, containing five grains of iodoform, are ordered twice or thrice daily. In the chronic forms of this complaint, suppositories of tannin, or of iodoform, or long tampons of absorbent cotton, are employed, which have been dipped in astringent solutions of acetate of lead and of zinc to which laudanum has been added.—*Ibid.*

**Psoriasis.**

To clean away the scales and prepare the skin for tarry remedies, etc., in psoriasis, nothing, says Dr. Priessman, of Vienna, equals this :

℞

Salicylic acid, 6 grams.
Alcohol, 100 grams.

Rub moderately the affected part with this mixture, on some charpie.

---

**Fissure of the Rectum.**

Dr. Goodell treats fissures in two ways, viz: 1st, by cutting through the adjacent muscular fibres; and 2d, by overstretching the sphincter ani muscle. He much prefers the second method. To do this he inserts his two thumbs into the rectum, and pulls them apart until the sphincter begins to yield or he feels the rami of the ischia on each side. To do this requires the exercise of considerable force.

---

**The Treatment of the Funis.**

As soon as the child cries lustily, the cord is cut, and, the umbilical portion being firmly held by the thumb and forefinger, the free end is "stripped" of Wharton's jelly and of any blood that may remain in it. Any blisters of Wharton's jelly which still remain are emptied by this process of "stripping," are nicked, and their contents squeezed out. After the removal of the pressure of the thumb and forefinger all bleeding usually ceases, and then the cord is tied. No subsequent dressing is thereafter used, for the cord rapidly dries without smell and drops off without leaving a sore behind.—*Med. Record.*

---

**Diarrhœa.**

The London *Medical Record* informs us that Dr. Schorstein, of Vienna, advises the application of a douche of hot water, under strong pressure, to the umbilical region, in cases of diarrhœa. The temperature is at first 50°, but may be raised to 72°. The duration of the application lasts from three to five minutes ; after it the patient takes a hip bath of 50° to 62°. This treatment is generally repeated not more than twice daily. Dysenteric diarrhœa, combined with tenesmus, and dysentery itself, if not inveterate, are treated in the same way. The effect is very rapid, and lasts much longer than opium treatment does ; the pain is also calmed very quickly. The author has also found this hot douche answer in cases of colic caused by biliary calculus, many kinds of neuralgia, sciatica excepted, where it was desirable to remove renal calculi and gravel, or long accumulated fecal matter.—*Med. and Surg. Reporter.*

---

**Cough Mixture.**

The following has proved very efficient :

℞

Ext. juglandis cin. fl.,
Tinct. opii camphoratæ, āā ℥ ss.
Syr. Tolutani, ℥ i.
Syr. Pruni Virg., ℥ ij.

M. A teaspoonful occasionally. A fl. oz. of fl. ext. cubebs may be added with directions to shake the bottle.—*A. B. Weymouth.*

---

**Cutaneous Eruptions by Chloral.**

In *La France Medical*, Dr. Mayer records five cases in which the administration of chloral in affections of various nature was followed by definite symptoms in uniform order. These came on after eating, beginning with redness of the face, excited action of the heart, and dyspnœa. The redness spreads to the neck ; the palms, and sometimes the soles, being also affected. The eruption then appears on other parts of the body, and especially on the dorsal surface of the hands and wrists, the superior and anterior part of the thorax, the extensor surfaces of the knees and elbows, and the dorsum of the foot. It lasts from half an hour to a few hours, with itchiness of the part affected, and is followed the next day by slight desquamation. The spots are of a deep rose color, are sometimes slightly elevated, and have sometimes a sinuous border.—*Med. and Surg. Reporter.*

---

**Whooping Cough.**

Dr. J. J. Caldwell's mode of treating these cases is to place a steam atomizer in position on a table before the patient, charged with the following mixture :

℞

Ext. belladonnæ fluid, gtt. vi.—xij.

Ammonii bromidi, ℈i.

Potassii bromidi, ℈ij.

Aquæ destillat, fl. ℥ ij.

Mix. This spray is rapidly carried over into the face, mouth, and lungs of the child, and applied ten to fifteen minutes, until the pupils are dilated by the effects of the belladonna mixture ; the application to be made morning, noon, and bedtime. This has, so far, cut short the spasmodic cough within two or three days uniformly, and almost to a certainty.—*St. Louis Med. and Surg. Journal.*

**Ice Cream and Beef Juice.**

Dr. J. J. Tucker, in the *Chicago Journal of Medicine*, commends the following as an excellent dietary article :

℞

Cream, 120 grammes.
Sugar, 30 grammes.
Extract of vanilla, 8 grammes.
Beef juice, 8 grammes.

Any confectioner can make it, or it may readily be prepared at home with a freezer.

---

**Nervous Prostration.**

Dr. Andrews, of New York, recommends, especially for nervous and debilitated females:

℞

Acid. phosphorici dil., 1 ounce.
Elix. calisayæ, 4 ounces.
Elix. val. ammon, 2 ounces.
Glycerinæ, 3 ounces.
Vini xerici, 6 ounces.

M. S. One-half to one ounce three or four times a day.

---

**Reptile Poisons.**

A correspondent of the *Medical Brief* says:

I send you a recipe for bites of reptiles, spiders, and all poisonous insects. Give the patient about one gill of strained honey every ten or fifteen minutes, until vomiting is produced, and you will see the swelling subside, and your patient will get almost instant relief. It is especially recommended in the bites of rattlesnakes, moccasins, and all snake bites.

[We take no "stock" in the above.—ED. BISTOURY.]

---

**Puerperal Fever.**

Is invariably treated by intra-uterine injections of a warm two per cent. solution of carbolic acid. Ten-grain doses of quinia are given every four hours until marked cinchonism is produced. Morphia is administered in doses sufficiently large and as frequently as necessary to relieve pain. The whole surface of the abdomen is painted with the compound tincture of iodine, and covered with a large mush-poultice. If it is deemed necessary to open the bowels, large doses of calomel are used.—*Dr. Goodell.*

---

**Fungous Vegetations of the Endometrium.**

In fungous vegetations Dr. Goodell removes the unhealthy growth either by the dull or by the sharp curette, or, if the os is sufficiently patulous, by means of a small fenestrated polypus-forceps. The uterine cavity is then cleansed with a saturated tincture of iodine, provided the cervical canal is narrow. But when ti gapes, he prefers Monsel's solution. In the former condition he avoids the use of the iron, because it forms clots which cannot easily be expelled by the womb without giving rise to much pain. He deems the iron, however, the more efficacious treatment of the two.

---

**Cancer of the Cervix.**

Whenever practicable, the whole cervix is removed by either the hot or the cold wire. If this cannot be done, he removes the malignant growth by scraping, or by means of the gouge-forceps, and the surface is subsequently charred with the thermo-cautery. This radical treatment is reinforced by subsequent applications of the ethylate of sodium. In these operations upon the cervix, Dr. Goodell finds that injections of ordinary vinegar form an excellent means of controlling any embarrassing bleeding. By these means he has succeeded in *curing* several cases.

---

**Cardiac Palpitations.**

Dr. N. S. Davis, of Chicago, in a "Report on Drugs and Medicines to the Illinois State Medical Society," May, 1878, says :

"The reading of a paragraph in some medical publication concerning the influence of the fluid extract of the cactus grandiflora, or night-blooming cereus, over cardiac palpitations, led me to a clinical test of its medicinal virtues during the past year. The first case in which it was given was that of a female, aged about 28 years, whose nervous and digestive functions had been greatly impaired by the habitual use of morphine and alcoholic preparations. One of her most distressing symptoms while endeavoring to recover from the effects of her habit was cardiac palpitation, more especially during the early morning hours. From three to five minims of this fluid extract, given from four to six times in the twenty-four hours, afforded her great relief. Another patient was a man of good habits, but affected with chronic enlargement of the cervical glands, and apparently also of those along the bronchial tubes, and frequently paroxysms of suddenly recurring palpitations, and with feelings of suffocation whenever attempting to take a recumbent position. He has been using the fluid extract of the cactus grandiflora about two weeks, with decided benefit in keeping the action of the heart more regular. It appears to exert a quieting influence over cardiac excitability, without any visible effect on the functions of the stomach or excretory organs. Although at present very expensive, it is certainly worthy of further investigation.—*Virginia Med. Monthly.*

**Habitual Constipation.**

The following prescription is a very favorite one with Dr. Goodell :

℞
Ext. colocynth. comp., gr. ij.
Pulv. rhei., gr. j.
Ext. Belladonna, gr. $\frac{1}{4}$.
Ext. hyoscyami, gr. ss.
M. divide in pil. No. 1.
S. To be taken at bedtime.

In some cases $\frac{1}{16}$ of a grain of strychnia is added with profit to the foregoing. In most cases "*massage*" is employed with the very best results.

**Bronchitis.**

Dr. Bell says, The eucalyptus globulus has remarkable anti-catarrhal virtues. The only preparation which I have used has been the tincture prepared by several of our most eminent druggists in Edinburgh, and I have seldom prescribed more than a teaspoonful mixed with a wineglassful of water twice a day. In several cases of bronchitis, with profuse expectoration, I have witnessed remarkable benefit after a very brief use of the remedy, evinced by a rapid diminution of the discharge, and also by corresponding improvement in the general condition of the patient.—*Med. Gazette.*

**Ovariotomy.**

In six cases of ovariotomy performed within the past nine months, with but one death, the following procedure was invaribly adopted. A five per cent. solution of carbolic acid was used in the spray, and all instruments and sponges were immersed in a solution of the same strength. The pedicle was treated by the intra-peritoneal method, being transfixed, tied, and dropped within the abdominal cavity. The peritoneum was invariably included in the stitches which closed the abdominal wound. All obstinate bleeding points were tied with gut ligature, but the pedicle itself was secured by fine carbolized silk. In three cases where the adhesions were numerous, the glass drainage-tube was employed. The dressing consisted merely of salicylated cotton held in place by adhesive strips, the whole being secured by an elastic flannel binder. Dr. Goodell prefers the above dry applications to Professor Lister's wet dressing. The after-treatment consisted in opium enough to allay pain, and in one tablespoonful of milk combined with limewater given every three hours for the first forty-eight hours. As soon as flatulence escaped from the bowel the supply of food was increased. The patients were prepared for the operation by a soap-bath on the previous evening, and by the adminstration of one grain of opium at bedtime. On the following morning one grain of opium and ten grains of quinia were given. Dr. Goodell always operated at eleven o'clock in the morning, as being the time when the vital forces are at their best. When high temperature ensued, it was reduced by the application of an ice-cap, which was found to be an efficient means of lowering bodily heat.

**Perimetritis.**

Dr. Goodell has the patient put to bed, and kept quiet. Fly blisters are then applied locally over the abdomen. A series of these blisters are used. Together with this local treatment, $\frac{1}{24}$ of a grain of the bichloride of mercury, with ten grains of the muriate of ammonia, is given thrice daily in the mist. glycyrrhizæ comp.

The following mixture is also often used:

℞
Hydrarg. Chlo. corros, gr. j.
Liq. chlo. arsenitis, f ʒ ss.
Mist. ferri chlo.,
Acid. Muriat. dil., āā f ʒij.
Syrupi, f ℥ iij.
Aquæ, q. s. ad f ℥ vj.
M.
S. One tablespoonful after each meal.

**Nitrate of Amyl.**

Dr. L. P. Yandell says that Dr. B. W. Richardson is using nitrate of amyl internally in cases similar to those for which he recommended its inhalation. He commences with three-drop doses, gradually increasing until the desired effect is produced. He gives it in alcohol and glycerine, say:

Alcohol, ʒ iij.

Glycerine, ʒ v.

Amyl nitrate, gtt. xxiv.

He cannot see how it will be of service in chloroform-poisoning or in sea-sickness, although he has had no experience with it in such cases, but, Dr. Yandell adds, "How lovely it does act in spasmodic affections."

**Prolapse of both Ovaries,**
is best managed by the knee-breast posture, and by the administration of such alteratives as tend to lessen the congestion of those organs. Among these he deems the best to be the chloride of ammonium in combination with the bichloride of mercury. Sometimes, however, large doses of the bromide act very happily. He considers this dislocation to be due in a great measure to the congestion of the sexual organs from the use of measures to prevent conception. He finds that pessaries are rarely useful in this distressing condition. But, if any are employed, he considers Cutter's bulb to be the best. He regards this conditionas a very frequently overlooked cause of many pelvic aches and pains, which are attributed very generally to the uterus alone.

---

**The Diagnosis of Ovarian Cyst.**

Dr. Goddell distinguishes ovarian cyst from dropsy in the following manner: In a case of ascites, the abdomen, when the patient is placed on her back, is flat on top, and bulges out at the sides. In ovarian cyst, the very opposite is true. In ascites, the intestines float up to the top, and resonance is elicited upon percussion. In ovarian cyst, percussion gives only flatness.

There is one certain way of settling the question of the existence or non-existence of ovarian cysts, and that is by means of the aspirator. The fluid of ascites is straw-colored and limpid; that of a monocyst is perfectly clear and limpid, like spring-water; that of a polycyst is thick, dark, and turbid, from disintegrated red blood-corpuscles; that of an oligocyst is usually of a milk-and-water or of a light brown color. The discovery of the Drysdale ovarian cell in an aspirated fluid is proof positive of its origin.

---

**Cystitis in the Female.**

Transient cystitis, dependent upon obscure causes, is treated by rectal suppositories containing one grain each of the aqueous extract of opium and of the extract of belladonna. Hysterical cases generally yield to "massage" and electricity. In obstinate cases, Dr. Goodell warmly advocates the dilatation of the urethra throughout its whole extent by the introduction of the forefinger. In the therapeutical treatment of this troublesome disorder atropia is the most efficient remedy, and it may be combined with alkalies or acids, according to the condition of the urine. Injections of two-grains solution of quinia into the bladder, together with large doses of the same by the mouth, will often improve the condition of the patient. In very bad cases, perhaps the most efficient injection is one of the nitrate of silver, beginning with weak solutions and increasing their strength daily until twenty grains to the ounce solutions are tolerated. These strong solutions should not be allowed to remain in the bladder longer than ten or fifteen seconds. All malpositions of the womb must, of course, be rectified, especially if they have any bearing on the disease.

---

**Diarrhœa.**

In the London *Medical Times and Gazette*, Dr. B. Fieholson recommends, in ordinary diarrhœa and such as precedes dysentery, the following, which he finds almost universally successful:

℞

Plumbi acetatis, gr. iv.

Pulveris opii, gr. ¼.

M. For one dose, thrice daily.

No good, but rather disadvantage, resulted from increasing the amount of opium, though the quarter of a grain was decidedly useful. As to the acetate of lead, it is convenient and portable, sedative as well as astringent.

---

**Infantile Colic.**

Instead of treating these cases with opiates, Dr. J. P. F. Brunner recommends, in the *Pacific Medical Journal*, the following combination, which he finds gives almost instantaneous relief and effects a permanent cure:

℞
Tincturæ assafœtidæ, gtt. xv.
Tincturæ cinnamomi, ℥ ss.
Sodii bicarbonatis, ʒ j.
Syrupi rhei aromat, ʒ iij.
Aquæ, ℥ iss.
M. Sig.—Half a teaspoonful every three hours.

---

**Experiments on the Living Brain,**

As an illustration of the perfection to which physiological experimentation has been brought, we submit the following item which we see going the round of the medical press, Prof. Wilder has no superior in this department of science. His cabinet of brain preparations is one of the finest in the world. But here is the item referred to:

"At the close of one of the daily sessions of the American Association for the Advancement of Science, Prof. Burt G. Wilder gave illustrations of some of the experiments of Ferrier on the brains of living animals. Having by the use of ether reduced a cat to the state of insensibility, Professor Wilder laid bare the surface of the animal's brain by removing the roof of the skull. On the wall was hung a diagram of the brain, with certain regions of it designated by figures. A chart stated what movements would be made by the cat, as these different regions of its brain were successively touched by the terminals of a weak electric current, and in every case the movements occurred precisely as laid down in the chart. Thus, when the place on the brain answering to that marked "1" in the diagram was touched, the opposite hind leg of the animal was advanced as the chart said it would be. When "4" was touched the opposite fore leg moved as if to strike, being first drawn back. Again, the animal was made to scream, spit and lash its tail, by similar means.

**Baldness.**

The following is highly recommended by Professor Kaposi :

℞
Saponis viridis, (German) alcoholis, āā f. ℥ ij.
Solve filtra, et adde ol.
Lavandulæ, gtt. xx.-xxx.

Pour one or two tablespoonfuls upon the scalp ; then pour on a little water ; rub smartly with the fingers, thus producing a copius lather. After four or five minutes' shampooing this way, rinse the head thoroughly with pure water, and dry thoroughly with a towel. Then apply a little vaseline. This process causes the hair to fall out in greater abundance at first, but a new and fine growth of hair soon follows.

**Prize for Essay on Diphtheria.**

The Empress of Germany has offered a prize of 2,000 marks ($500) for the best essay on diphtheria. The conditions are that the writer is to bring forward important *new* facts as to the essential nature (*das Wesen*) of the disease, especially with regard to the infectious matter which propagates it, its dissemination, and the means for arresting its progress. The essays may be written in German, English or French, and must be sent to Profesfor v. Langenbeck, Berlin, N. W., 3 Roonstrasse, on or before December 15, 1880. The committee which will award the prize consists of Professors Klebs of Prague, Liebreich and Virchow of Berlin, Von Nageli and Oertel of Munich, and Thiersch of Leipsic. Each essay is to have a motto corresponding to a similar motto on a sealed envelope containing the author's name.

**Whooping Cough Syrup.**

Roasted coffee, 16 ounces.
Boiled water, sufficient.
Extract of Belladonna, 2½ drachms.
Extract of ipecac, 2½ drachms.
Sugar, 62 ounces.

Exhaust the coffee with enough boiling water to obtain about thirty-four ounces of percolate. In this dissolve the extracts, and lastly the sugar.

Dose, half an ounce morning and noon, and one ounce at night, mixed with two or three spoonfuls of warm water for children from three to five years old. For younger infants, half the quantity.

**Insomnia.**

In the treatment of insomnia it is important to first ascertain its cause. Slight cases are usually successfully treated by general hygienic measures.

Insomnia occurring during acute or chronic maladies, cannot, as a rule, be rapidly relieved. Therefore, while waiting the recovery of the disease, the symptom is to be treated with hypnotics, at the head of which is opium and its alkaloids.

Morphia is the most somniferous principle of opium. Narcein and codeine, although less active in this respect, leave fewer traces of headache and malaise. Opium preparations are more particularly useful in insomnia associated with pain. They are contra-indicated where there exists any cerebral congestion.

Bromide of potassium has a much less powerful hypnotic action than opium. Its use is indicated in those cases due to excitement of the cerebral circulation, in which opiates are useless and injurious. It has been employed successfully as a calmative in children. It is contra-indicated in cases of marked anæmia.

Sulphate of quinine, like the bromide, appears to exercise the action of relieving the congestion of the cerebral nervous elements.

Hydrate of chloral is an excellent hypnotic in almost all cases of insomnia, but it is to be given with caution to persons suffering from dyspnœa, cardiac affections, or great debility.

The insomnia of old persons or patients suffering from great debility or anæmia is sometimes successfully treated by tonics, stimulants, and hydropathy.—*Medical and Surgical Reporter*.

**Whooping Cough.**

Dr. Garth (*Wiener Allgem*) states that by placing xx. gtt. ol. terebinth on a handkerchief, holding it before the face, and taking about forty deep inspirations, to be repeated thrice daily, signal and marked relief, followed by rapid cure in cases of laryngeal catarrh, is the result. In an infant fifteen months old, in the convulsive stage of whooping cough, he directed the mother to hold a cloth, moistened as above, before it when awake, and to drop the oil on the pillow when asleep. The result was markedly beneficial. In twenty-four hours the frequency and severity of the attacks were notably diminished, and by proper support, by aid of stimulants, the improvement was rapid. Subsequently pertussis became epidemic in his vicinity, and he repeatedly used the drug in this way. He gave it to children of all ages, and in any stage of fever. The initial catarrh, the convulsive, and the final catarrhal stages were all decidedly benefited, the spasmodic attacks being in many cases aborted.

**Test for Diabetic Urine.**

It is almost an every day's occurrence for the physician to have to ascertain the presence or absence of sugar in a patient's urine. Many practitioners, owing to the lack of the necessary apparatus and reagents, often send the suspected liquid to a pharmaceutist or a chemist for examination.

The object of a note read by Dr. Leon Fredericq before the Medical Society of Ghent (Belgium), is to call the attention of physicians to a simple method of detecting sugar in urine by means of the fermentation observed when glucose is brought into contact with beer yeast.

The process recommended is as follows: First, the subjoined objects are to be procured: (1) An ordinary medicine vial of the capacity of about five ounces, provided with a common cork. A small groove or slit is to be cut lengthwise with a penknife in the cork, so as to leave in it a vertical opening; (2) a large tumbler half filled with water; (3) a piece of beer yeast, still moist, about the size of a walnut.

The yeast may be broken up and introduced in small fragments into the vial, or better still, rubbed with water into a semi-liquid paste, which is poured into the bottle. The vial is next *completely* filled with the urine, and the cork adjusted, care being taken that the slit remains open. The bottle is then turned upside down into the tumbler, its neck resting on the bottom, and the cork being well under water.

The whole is left exposed to a gentle heat, in the sun or near the fire in winter, a temperature of 77° F. being the most favorable to the reaction. If the urine contains sugar, the fermentation proceeds rapidly, and bubbles of carbonic acid gas form and accumulate in the upper part of the vial. In measure as the gas increases in quantity, the liquid is forced from the bottle, and escaping through the little conduit provided in the cork, mixes with the water in the tumbler. After a short time a notable portion of the bottle is filled with gas. The fermentation proceeds for a couple of days longer, and in the end, if the sugar is present in a certain proportion, the whole bottle is found to contain nothing but gas, while all the liquid has been expelled.

The reaction is unmistakable and readily produced. It also presents other advantages. The use of cupro-potassic or bismuthic reagents occasionally leads to erroneous results. It may happen that urine containing no sugar reduces the blue cupric solution. After the administration of chloral, for instance, urine is found to contain a reductive substance which is not glocuse. On the other hand, certain diabetic urines fail to afford the characteristic cuprous precipitate. This is easily understood, since the chemical reaction by which it is thus attempted to detect glucose is not one peculiar to it, but one applicable to all reductive substances. The same reaction is, besides, hindered or prevented by the presence of certain bodies, among which creatinine has been mentioned.

The fermentation of sugar by means of yeast, on the contrary, is much more characteristic, more special, and as such less liable to error. To this process it might be objected that commercial yeast occasionally contains sugar, but it is in too small a proportion to be a serious cause of error. It is, besides, easy to eliminate the possible error by either repeating the experiment with normal urine, or using only yeast which has previously been washed in water.

